# Prevalence and Prognosis of HFimpEF Developed From Patients With Heart Failure With Reduced Ejection Fraction: Systematic Review and Meta-Analysis

**DOI:** 10.3389/fcvm.2021.757596

**Published:** 2021-11-25

**Authors:** Yibo He, Yihang Ling, Wei Guo, Qiang Li, Sijia Yu, Haozhang Huang, Rongting Zhang, Zhiwen Gong, Jiaxuan Liu, Liyi Mo, Shixin Yi, Disheng Lai, Younan Yao, Jin Liu, Jiyan Chen, Yong Liu, Shiqun Chen

**Affiliations:** ^1^Department of Cardiology, Guangdong Provincial Key Laboratory of Coronary Heart Disease Prevention, Guangdong Cardiovascular Institute, Guangdong Provincial People's Hospital, Guangdong Academy of Medical Sciences, Guangzhou, China; ^2^Guangdong Provincial Geriatrics Institute, Guangdong Provincial People's Hospital, Guangdong Academy of Medical Sciences, Guangzhou, China; ^3^Department of Cardiology, Longyan First Affiliated Hospital of Fujian Medical University, Longyan, China; ^4^Department of Cardiology, First People's Hospital of Kashgar Prefecture, Kashgar, China; ^5^Department of Cardiology, The Fifth Affiliated Hospital of Sun Yat-sen University, Zhuhai, China

**Keywords:** heart failure, recovered or improved ejection fraction, mortality, hospitalization, meta-analysis

## Abstract

**Background:** Heart failure with improved ejection fraction (HFimpEF) is classified as a new type of heart failure, and its prevalence and prognosis are not consistent in previous studies. There is no systematic review and meta-analysis regarding the prevalence and prognosis of the HFimpEF.

**Method:** A systematic search was performed in MEDLINE, EMBASE, and Cochrane Library from inception to May 22, 2021 (PROSPERO registration: CRD42021260422). Studies were included for analysis if the prognosis of mortality or hospitalization were reported in HFimpEF or in patients with heart failure with recovered ejection fraction (HFrecEF). The primary outcome was all-cause mortality. Cardiac hospitalization, all-cause hospitalization, and composite events of mortality and hospitalization were considered as secondary outcomes.

**Result:** Nine studies consisting of 9,491 heart failure patients were eventually included. During an average follow-up of 3.8 years, the pooled prevalence of HFimpEF was 22.64%. HFimpEF had a lower risk of mortality compared with heart failure patients with reduced ejection fraction (HFrEF) (adjusted HR: 0.44, 95% CI: 0.33–0.60). HFimpEF was also associated with a lower risk of cardiac hospitalization (HR: 0.40, 95% CI: 0.20–0.82) and the composite endpoint of mortality and hospitalization (HR: 0.56, 95% CI: 0.44–0.73). Compared with patients with preserved ejection fraction (HFpEF), HFimpEF was associated with a moderately lower risk of mortality (HR: 0.42, 95% CI: 0.32–0.55) and hospitalization (HR: 0.73, 95% CI: 0.58–0.92).

**Conclusion:** Around 22.64% of patients with HFrEF would be treated to become HFimpEF, who would then obtain a 56% decrease in mortality risk. Meanwhile, HFimpEF is associated with lower heart failure hospitalization. Further studies are required to explore how to promote left ventricular ejection fraction improvement and improve the prognosis of persistent HFrEF in patients.

**Systematic Review Registration:** https://www.crd.york.ac.uk/prospero/display_record.php?ID=CRD42021260422, identifier: CRD42021260422.

## Introduction

Heart failure (HF) is a significant cause of cardiovascular disease death and rehospitalization, which tends to be a major socioeconomic burden (1, 2). Left ventricular ejection fraction (LVEF) is widely used as an important indicator for classification and prognosis in patients with heart failure, of which a cut-off point of lower than 40% was defined as reduced ejection fraction (HFrEF) (3). Due to medical treatment or natural recovery of heart failure, the increase of ejection fraction was found in a portion of HFrEF patients during follow-up. Punnoose et al. identified a subset of heart patients with preserved ejection fraction (HFpEF) recovered from a previously reduced ejection fraction (4). Several subsequent studies had found that patients with heart failure with improved ejection fraction (HFimpEF) or recovered ejection fraction (HFrecEF) were novel clinical entities and significantly different from HFrEF and HFpEF (5–7). For this current situation, the Heart Failure Society of America (HFSA), Heart Failure Association of the European Society of Cardiology (HFA/ESC), and the Japanese Heart Failure Society (JHFS) published the latest consensus statement of a universal definition for HF. HF with a second measurement of LVEF > 40% and a ≥10% increase from baseline LVEF of ≤ 40% was defined as HFimpEF (8), a more proper definition that implies not a full recovery in cardiac structure and function despite improvement in EF, which used to be classified as HFrecEF.

Previous HFrEF patients who developed HFimpEF during the follow-up visit were demonstrated with not only a better prognosis but also a significant improvement in health-related quality of life (6, 9). However, different conclusions appeared in Joan Carles Trullàs's study, which showed that the risk of death between HFimpEF and HFrEF groups was not statistically significant (10). At present, there is no universal understanding of the association between HFimpEF and the prognosis. Additionally, the prevalence of HFimpEF or HFrecEF was diverse in different studies. Considering these inconsistent findings at present, a systematic review of the prevalence and prognosis of patients with HFimpEF or HFrecEF is important and urgently needed.

Therefore, we conducted a systematic review and meta-analysis of published studies to obtain a comprehensive quantitative assessment of prevalence and prognosis (e.g., mortality) of the patients with HFrEF, who eventually developed HFimpEF or HFrecEF.

## Methods

Studies that reported mortality and hospitalization outcomes of patients with HFimpEF, including patients with heart failure with improved or recovered ejection fraction, were eligible for the systematic review and meta-analysis. The primary outcome was follow-up mortality, and the secondary outcomes included heart failure hospitalization, all-cause hospitalization, and composite endpoints of death and hospitalization The study was reported in accordance with the PRISMA (preferred reporting items for systematic reviews and meta-analysis) statement. The study protocol was registered on PROSPERO (CRD42021260422).

A comprehensive strategy was applied in the literature search on MEDLINE, EMBASE, and Cochrane Central databases from inception to May 22, 2021. The keywords of the search included heart failure AND recovered ejection fraction OR improved ejection fraction (see details in the Supplementary Material). We included studies that reported detailed data of risk in patients with heart failure with improved or recovered ejection fraction. No restriction was applied to the language of studies. However, if studies were classified as review articles, case reports, conference abstracts, comments or editorial, animal studies, they would be excluded from the screening.

Screening on titles and abstracts of the collected studies was performed by reviewers (JL, RZ, ZG, and LM) independently according to eligibility criteria. Disagreements were solved by the third reviewer (YH) after careful review. YH and WG performed independent data extraction through a full-text review. Baseline characteristics and outcome data were extracted, including author, publication year, study country, study design, definition of recovered or improved ejection fraction, follow-up duration, male proportion, and median age. The hazard ratio of the outcomes was the target effect size used for synthesis. For studies which reported the prognosis of different follow-up periods, data of the longest follow-up visit was finally collected for analysis. Extracted data were double-checked by SC, and disagreements were resolved by discussion. Newcastle–Ottawa Scale (NOS) was applied to assess the quality of the included studies by QL and YL independently. Disagreements were resolved by group discussion until a consensus was made.

The statistical analysis was performed using R software (version 4.1.0). Pooled quantification was calculated to obtain the hazard ratio and 95% confidential interval. When studies demonstrated low or moderate heterogeneity, a fixed-effects model was applied; a random effect model was applied if the studies demonstrated high heterogeneity. *I*^2^ statistic was calculated to evaluate the heterogeneity among studies. *I*^2^-valued 0–25% was considered low heterogeneity, whereas 25–50% and over 50% values represented moderate and high degrees of heterogeneity, respectively. We performed sensitivity analysis by omitting one study successively to evaluate the impact of the individual studies on the pooled effect size. A two-sided *p*-value of < 0.05 was considered statistically significant.

## Results

**Figure 1:**
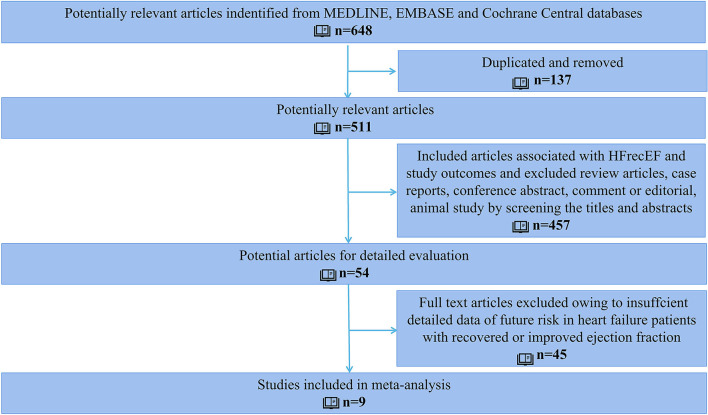
Flowchart of studies selection in the meta-analysis.

After screening 648 retrieved studies from the systematic search, 54 records met eligibility criteria. After the full-text review, nine studies were finally included in the analysis (Figure 1).

The study involved 9,491 heart failure patients, of which 1,596 patients were found to have improved or recovered ejection fraction. Half of the studies were prospective design whereas the others were retrospective design. Five out of the nine studies defined HFimpEF as patients with previously documented EF <40% but recovered to over 40% during the follow-up visit. Two studies defined HFimpEF as an improvement from <50% to over 50%, one study defined HFimpEF as an improvement from <40% to over 50%, one study defined HFimpEF as an improvement from <45% to over 45%. The average prevalence of HFimpEF was 22.64% (range from 10.36 to 52.07%) among the baseline HFrEF patients. Details of the study characteristics are shown in Table 1. Study quality assessed by the Newcastle–Ottawa scale demonstrated that two studies scored 7, one study scored 8, and the remaining studies scored 9, which indicated the good quality of the included studies (Table 2).

**Table 1:** Baseline characteristic of included studies reporting heart failure patients with improved or recovered ejection fraction.

**References**	**Region**	**Study period**	**Design**	**Study arms**	**Definition**	**Population sample**	**Incidence ratio of HFimpEF/HFrEF**	**Mean follow-up**	**Outcome**	**Male**	**Age**
Agra Bermejo et al. (11)	Spanish	September 2007 to January 2014	Retrospective	HFrecEF/ HFpEF/ HFrEF	(HFpEF) LVEF > 40% (HFrEF) LVEF 40% (HFrecEF) LVEF ≤ 40% Recovered to LVEF > 40%	449	126/242 (52.07%)	1,800 ± 900 days	Mortality; hospitalization rate	HFpEF: 120 (58%) HFrEF: 89 (76.7%) HFrecEF: 92 (73%)	HFpEF: 71 ± 10 HFrEF: 66 ± 12 HFrecEF: 63 ± 12
Basuray et al. (5)	USA	2003–2012	Prospective	HFrecEF/ HF-REF/ HF-PEF	(HF-REF) LVEF <50% (HF-PEF) LVEF consistently ≥ 50% (HFrecEF) LVEF ≥ 50% but prior LVEF <50%	1,821	176/1,699 (10.36%)	3.6 years	Mortality, transplantation or VAD (ventricular assist device) placement; hospitalization	HFrEF: 1,061 (70%) HFpEF: 56 (46%) HFrecEF: 94 (53%)	HFrEF: 56 (14) HFpEF: 63 (14) HFrecEF: 57 (13)
Chang (12)	USA	June 12, 2001 to July 19, 2004	Prospective	HFrecEF/ HFrEF	(HFrecEF) EF <35 to > 40% in 6 months (HFrEF) EF <40% at 6 month follow-up	318	59/318 (18.55%)	18 months	Mortality; first HF hospitalizations; recurrent HF hospitalizations; first all-cause hospitalizations; recurrent all-cause hospitalizations	HFrecEF: 35 (59.3%) HFrEF: 164 (63.3%)	HFrecEF: 55.7 + 11.8 HFrEF: 57.3 + 12.9
Kalogeropoulos et al. (6)	USA	January 1, 2012 to April 30, 2012	Retrospective	HFrecEF/ HFpEF/ HFrEF	(HFrEF)current LVEF ≤ 40% (HFpEF) current and all previous LVEF > 40% (HFrecEF)current LVEF > 40% but any previously LVEF ≤ 40%	2,166	350/1,700 (20.59%)	3 years	Mortality; hospitalization rates; composite endpoints (death or first hospitalization for any cause; death or first hospitalization for cardiovascular causes; and death or first HF-related hospitalization)	HFrEF: 887 (65.7%) HFpEF: 201 (43.1%) HFrecEF: 182 (52.0%)	HFrEF: 63 (51–72) HFpEF: 72 (62–82) HFrecEF: 65 (55–74)
Martínez-Mateo (13)	Spanish	January 1, 2010 to June 30, 2017	Prospective	HFrecEF/HFrEF	(HFrecEF) EF <40 to >50% at follow-up (HFrEF) EF <40%	431	116/431 (26.91%)	50 months	All-cause mortality; death for heart failure; cardiac death	HFrecEF: 79.3% HFrEF: 79.4%	HFrecEF: 64.3 ± 12.3 HFrEF: 68.0 ± 12.6
Nadruz (7)	USA	July 2007 to June 2013	Retrospective	HFmEF/ HFrEF/ HFm-recEF HFpEF	(HFrEF) LVEF <40% (HFmEF) LVEF was between 40 and 55% (HFpEF) LVEF > 55% (HFm-recEF) LVEF was between 40 and 55% but previously <40%	958	184/804 (22.89%)	4.4 years	Composite events (death, left ventricular assistant device implantation, or transplantation)	HFrEF: 452 (73%) HFm-recEF: 104 (61%) HFmEF: 59 (55%) HFpEF: 23 (49%)	HFrEF: 5.4 ± 13.2 HFm-recEF: 2.2 ± 13.0 HFmEF: 54.4 ± 15.2 HFpEF: 63.3 ± 15.5
Trullàs (10)	Spanish	March 2008 to September 2009	Prospective	HFrecEF/ HFrEF	(HF-PEF) LVEF ≥ 50% (HF-REF) LVEF persistently <50% (Rec-HF) LVEF > 50% and an absolute increase >5% from baseline LVEF <50%	1,202	27/108 (25%)	367 days	first readmission due to acute decompensation of HF; death by any cause	HFpEF: 441 (40%) HFrEF: 47 (58%) HFrecEF: 16 (59%)	HFpEF: 79.9 ± 8.0 HFrEF: 73.6 ± 10 HFrecEF: 71.6 ± 11
Wang et al. (14)	Canada	January 2009 to December 2019	Retrospective	HFrecEF/ HFrEF/ HFtrecEF/ HFpEF	(HFrEF) LVEF <40% (HFrecEF) baseline LVEF <40%, but improved to >40% and with a ≥10% improvement (HFtrecEF) recovery in LVEF from <40 to >40% and with a ≥10% improvement but back to <40% within the study period (HFpEF) LVEF <50%	1,089	325/806 (40.32%)	6.6 years	All-cause; Cardiovascular conditions; HF hospitalizations and mortality	HFrEF: 282/364 (77.5%) HFrecEF: 231/325 (71.1%) HFtrecEF: 96/117 (82.1%) HFpEF: 164/283 (58.0%)	HFrEF: 62 (54–71) HFrecEF: 57 (51–68) HFtrecEF: 61 (53–69) HFpEF: 68 (59–77)
Lupón et al. (15)	Spain	August 2001 to December 2015	Prospective	HFrecEF/ HFrEF/ HFpEF	HF-recovered: LVEF <45% at baseline and and mortalyear HFpEF: LVEF ≥ 45% throughout follow-up HFrEF: LVEF <45% throughout follow-up	1,057	233/940 (24.8%)	5.6 ± 3.1 years	Composite of cardiovascular death or HF hospitalization; all-cause, CV cause, HF-related, and sudden death, and the total number of HF hospitalizations.	HF-recovered: 164 (70.4%) HFpEF: 38 (32.5%) HFrEF: 573 (81.0%)	HF-recovered: 63.2 ± 12.4 HFpEF: 69.5 ± 13.8 HFrEF: 65.9 ± 11.3

**Table 2:** Newcastle–Ottawa scale scores and quality assessment of included studies.

	**Selection**					**Outcome**			
**References**	**Representativeness**	**Selection**	**Ascertainment**	**Outcome**	**Comparability**	**Assessment**	**Follow-up**	**Adequacy**	**Total score**
Agra Bermejo et al. (11)	*	*	*	*	**	*	*	*	9
Basuray et al. (5)	*	*	*	*	**	*	*	*	9
Chang (12)		*	*	*	*	*	*	*	7
Kalogeropoulos et al. (6)	*	*	*	*	**	*	*	*	9
Nadruz (7)	*	*	*	*	**	*	*	*	9
Trullàs (10)	*	*	*	*	*	*	*		7
Wang et al. (16)	*	*	*	*	**	*	*	*	9
Martínez-Mateo (13)	*	*	*	*	*	*	*	*	8
Lupón et al. (15)	*	*	*	*	**	*	*	*	9

*stands for 1 score.

**Figure 2:**
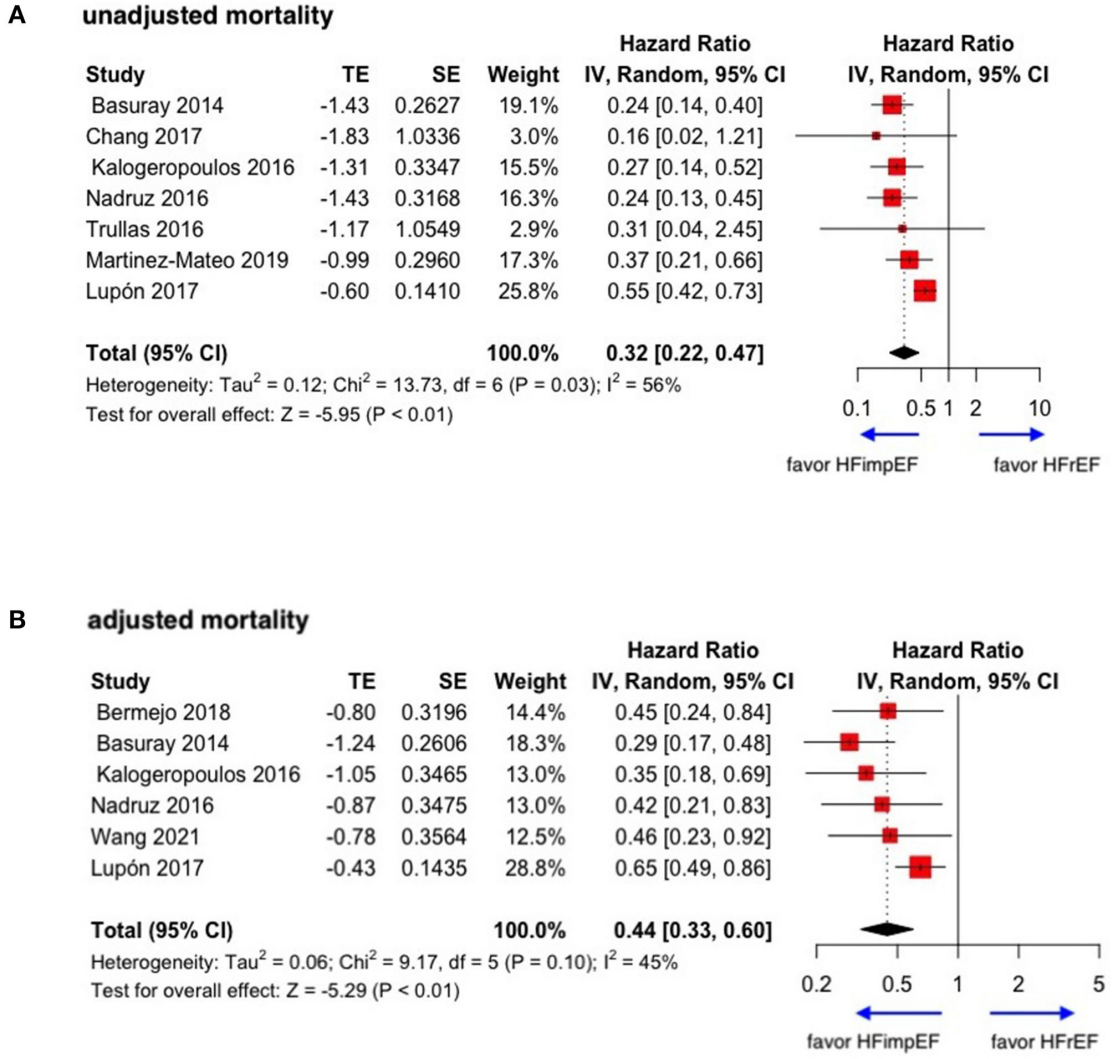
Forest plots of unadjusted and adjusted mortality in patients between HFimpEF and HFrEF. **(A)** Unadjusted mortality. **(B)** Adjusted mortality.

**Figure 3:**
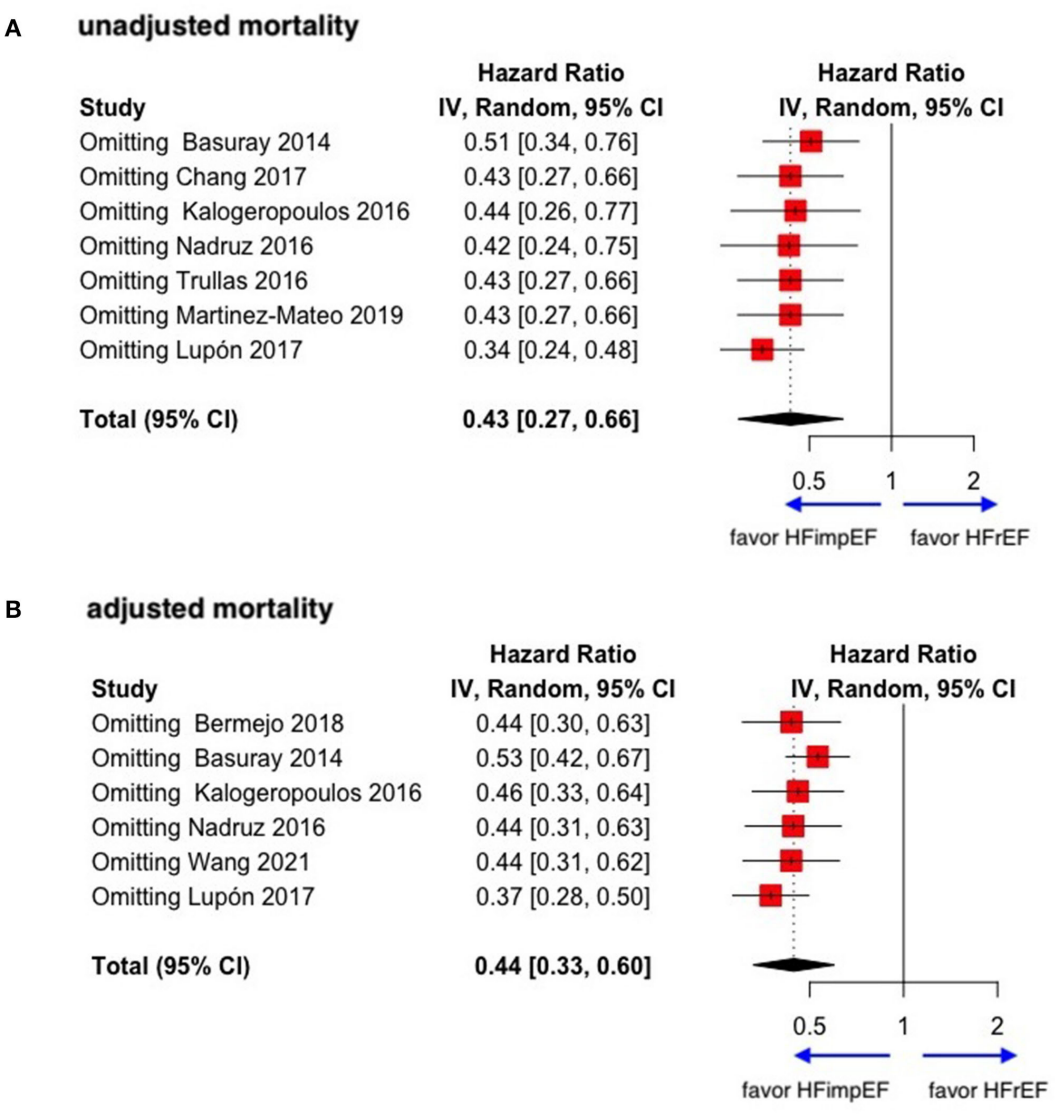
Sensitive analysis of unadjusted and adjusted mortality in patients between HFimpEF and HFrEF. **(A)** Unadjusted mortality. **(B)** Adjusted mortality.

**Figure 4:**
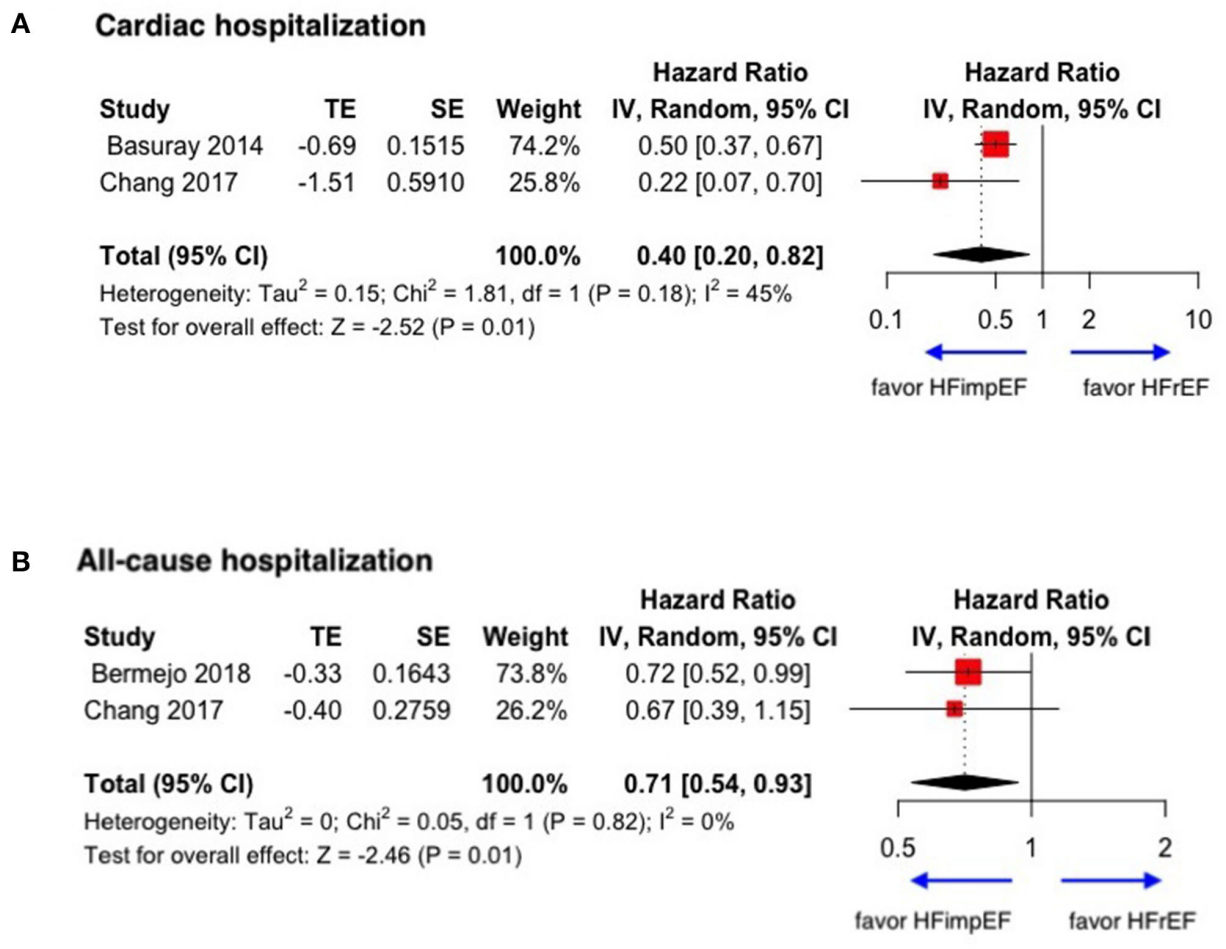
Forest plots of cardiac hospitalization and all-cause hospitalization in patients between HFimpEF and HFrEF. **(A)** Cardiac hospitalization. **(B)** All-cause hospitalization.

**Figure 5:**
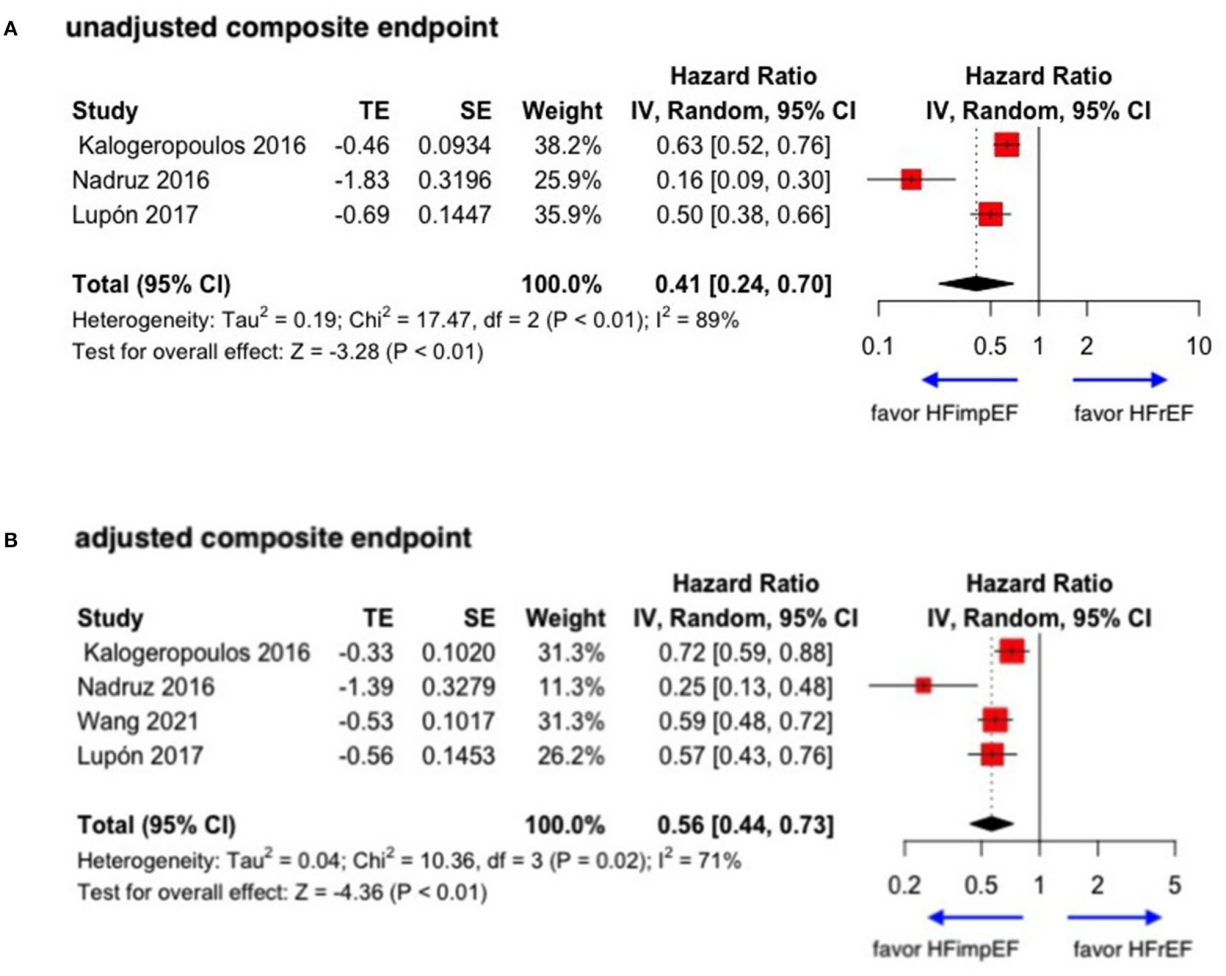
Forest plots of unadjusted and adjusted composite endpoint in patients between HFimpEF and HFrEF. **(A)** Unadjusted composite endpoint. **(B)** Adjusted composite endpoint.

During a median follow-up of 3.8 years, patients with heart failure with improved ejection fraction or recovered ejection fraction had a lower risk of follow-up mortality compared to patients with reduced or preserved ejection fraction (unadjusted HR: 0.32, 95% CI:0.22–0.47, adjusted HR: 0.44, 95% CI: 0.33–0.60) (Figure 2). When omitting one study successively to assess the sensitivity, the pooled effect size remained stable (Figure 3). As for hospitalization outcome, HFimpEF had 60% reduced risk of cardiac hospitalization (HR: 0.40, 95% CI: 0.20–0.82) and 29% had reduced risk of all-cause hospitalization (HR: 0.71, 95% CI: 0.54–0.93) compared with HFrEF patients (Figure 4). Overall, HFimpEF reduced the risk of the composite events of mortality and hospitalization by 44% (adjusted HR: 0.56, 95% CI: 0.44–0.73; unadjusted HR: 0.41, 95% CI: 0.24–0.70) (Figure 5).

**Figure 6:**
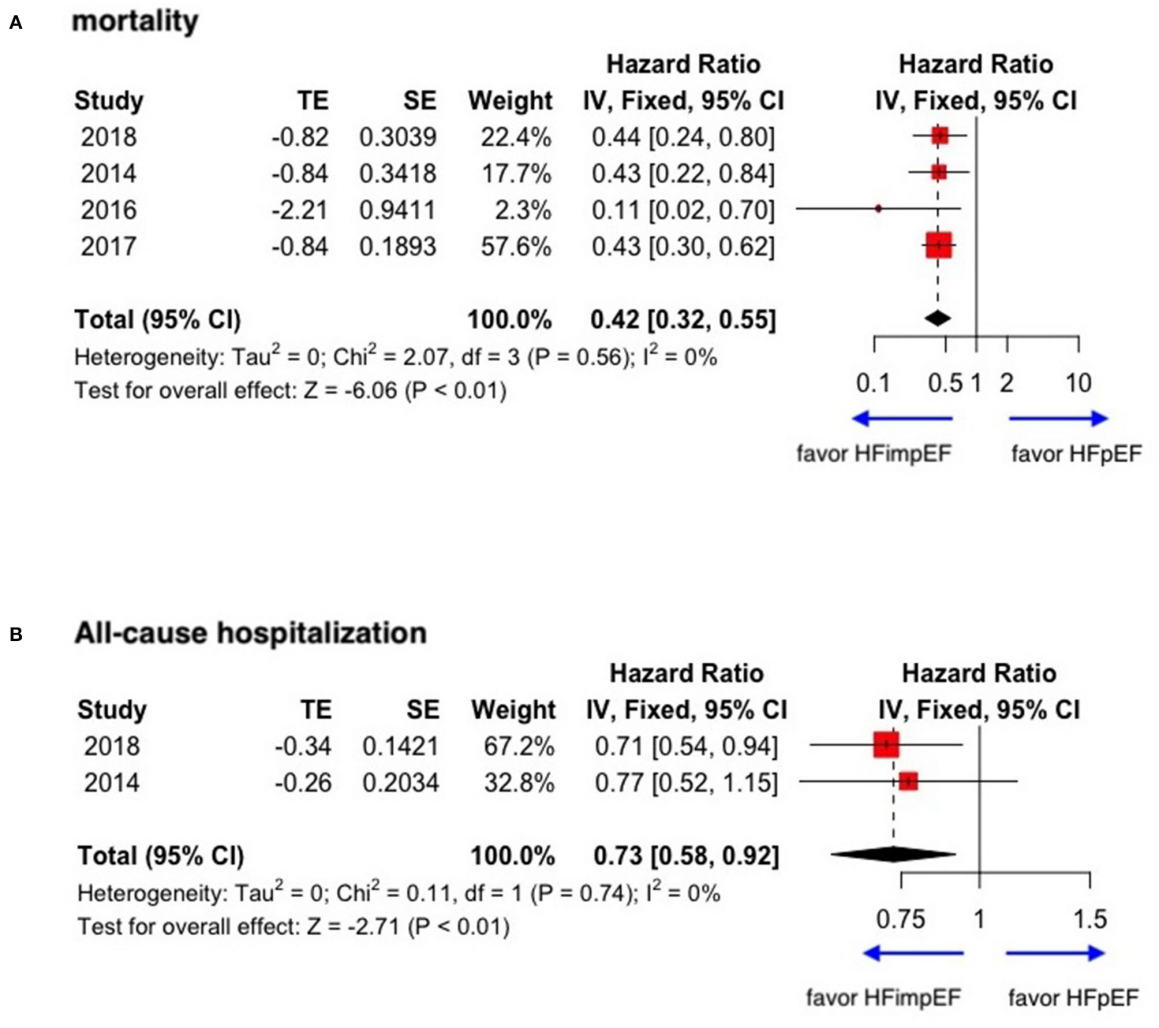
Forest plots of mortality and all-cause hospitalization in patients between HFimpEF and HFpEF. **(A)** Mortality. **(B)** All-cause hospitalization.

**Figure 7:**
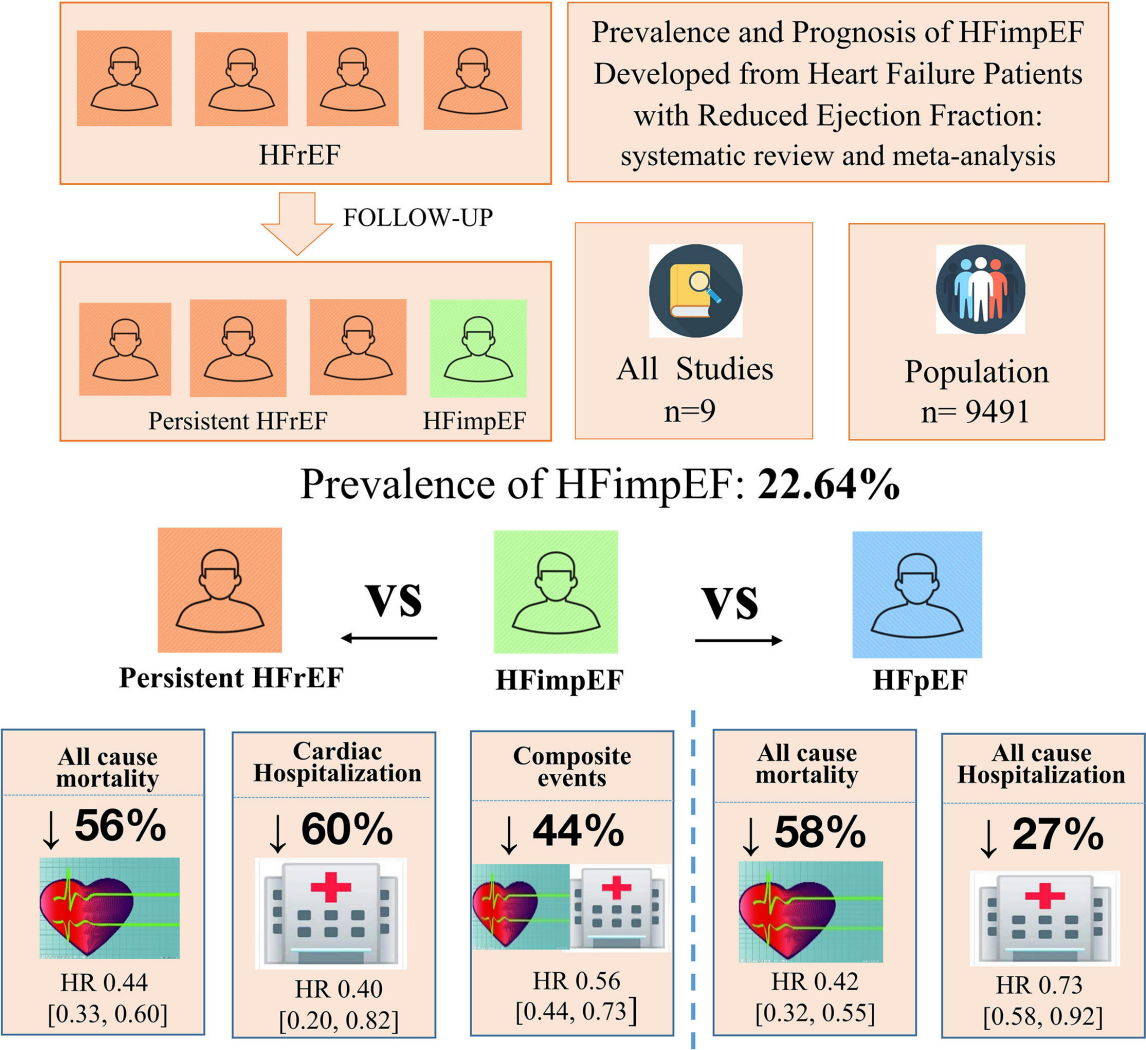
Concluded chart of the study results.

With limited data, HFimpEF patients were observed with a moderately lower risk of mortality (unadjusted HR: 0.42, 95% CI: 0.32–0.55) and all-cause hospitalization (HR: 0.73, 95% CI: 0.58–0.92) compared with HFpEF patients (Figure 6). The concluded results of the study are shown in Figure 7.

Minor or moderate heterogeneity was observed between studies regarding mortality and hospitalization between HFimpEF and HFrEF. However, the heterogeneity was prominent in the composite events. In studies comparing outcomes between HFimpEF and HFpEF, the heterogeneity ranged from 0 to 1%.

## Discussion

This is the first known systematic review and meta-analysis to evaluate the prevalence and prognosis of HFrEF patients who developed HFimpEF. Our study demonstrated that 22.64% of HFrEF would develop HFimpEF after treatment. HFimpEF was associated with a 56% decrease in mortality and a 60% decrease in cardiac hospitalization compared with HFrEF patients.

Left ventricular ejection fraction is an important indicator for the evaluation of symptoms and prognosis in patients with heart failure. After recommended treatment in current guidelines for heart failure, a portion of HFrEF patients were observed with improved ejection fraction value during follow-up visits, which may constitute a part of the growing number of HFpEF patients (5, 17). The use of evidence-based heart failure therapies in the outpatient setting improvement study reported that after 1 year of treatment, the average LVEF of patients with heart failure increased from 25.8 to 32.3% (18). Several studies have confirmed recovered or improved ejection fraction as an independent group associated with reduced adverse events, such as cardiovascular death and hospitalization, compared with both HFrEF and HFpEF patients (5, 6). In addition to the effect on mortality and hospitalization outcomes, Peter Wohlfahrt et al. confirmed that HFrecEF significantly improved the quality of life in patients with heart failure (9). However, the prognostic effect of recovered ejection fraction was inconsistent or even non-significant (10, 12, 16). After a systematic review of all relevant reports, we have pooled the quantified impact of HFrecEF on prognosis, which provided explicit evidence that HFrecEF, recently redefined as HFimpEF, is a novel entity in patients with heart failure needing more attention and evaluation.

The definition of HFrecEF is not consistent in various studies. The most universal definition of HFrecEF was the recovery of reduced ejection fraction to the level of preserved ejection fraction based on the specified definition in the studies. For example, Kalogeropoulos et al. defined HFrecEF as the recovery of ejection fraction from the level of reduced EF (below 40%) to preserved EF (above 40%) (6, 11), while Basuray defined HFrecEF as the recovery from below 50% (HFrEF) to above 50% (HFpEF) (5, 19). However, in the latest consensus, it was indicated that HF with a second measurement of LVEF > 40% and a ≥10% increase from baseline LVEF of ≤ 40% should be defined as HFimpEF (8), which implied that the change of ejection fraction in these patients would be better defined as improvement other than recovery to the level of preserved ejection fraction. Nonetheless, whichever definition was adopted, the HFimpEF was demonstrated to be associated with a better prognosis according to the results in our study. Cintron et al. (20) reported that even a minor improvement of 5% in ejection fraction was an independent predictor of survival. Therefore, it is indicated that the change or improvement of ejection fraction is associated with prognosis, rather than the level of ejection fraction. Dynamic detection of ejection fraction is necessary to evaluate the prognosis. Moreover, due to the minor gap of the EF change between the definition of HFimpEF and HFrecEF, further studies were warranted to better differentiate the effect of HFimpEF and HFrecEF on the following outcomes in patients with heart failure.

Reverse left ventricular remodeling with a more favorable neurohormonal profile is probably the main mechanism of HFimpEF or HFrecEF, which was characterized as the reduction of left ventricular end-diastolic and end-systolic volume, left ventricular mass index, and E/e′ ratio (5, 16). Kramer et al. had reported that reverse left ventricular remodeling is associated with fewer heart failure hospitalizations and reduced cardiovascular mortality, and the degree of reverse left ventricular remodeling is directly related to improved cardiac survival (21). Notably, reverse left ventricular remodeling was found to be a unique characteristic of HFrecEF patients, and the greatest magnitude of EF change was observed within 2 years following cardiac damage (22). On the other hand, a significant number of patients with heart failure were reported to experience recovered left ventricular function naturally, after elimination of myocardial injury caused by potential reasons of energetic abnormalities, toxic injury, and inflammation (23). For example, treatment of hyperthyroidism and hypothyroidism would be helpful for the recovery of LVEF (24). Timely reperfusion and revascularization are other reasons for the recovery of ejection fraction from ischemic etiology. It has been reported that patients with recovered ejection fraction had a lower incidence of coronary artery disease, and the absence of prior myocardial infarction and non-ischemic disease were both associated with an improved LVEF by more than 10% (5, 18). In addition, in patients with genetic heart failure, it had been reported that more than half of the patients with dilated cardiomyopathy and patients with hypertrophic cardiomyopathy would experience LVEF improvement after pro- per-medical treatment or cardiac resynchronization therapy, and the EF improvement was demonstrated to be associated with a lower risk of cardiac events as well (25–28). Moreover, restoration of LVEF has been reported to be associated with other characteristics of patients, such as younger age, female gender, left bundle branch block, and shorter duration of heart failure (4, 15, 18). However, the change of LVEF might not be linear and unidirectional that a patient may have improvement followed by a decline in EF or vice versa, depending on the underlying etiology, duration of disease, adherence to the medications, comorbidities, or reexposure to cardiotoxins (29).

To achieve improved or recovered ejection fraction, medications such as renin–angiotensin–aldosterone system and β-adrenoceptor blockers, recommended by international guidelines, were proven to be effective in heart failure treatment (3). Treatment with valsartan was associated with both reverse ventricular remodeling and LVEF recovery, yielding a better prognosis compared with HFrEF patients (30). In the KorAHF study, Park et al. (31) demonstrated that β-blocker were positive predictors of HF with improved ejection fraction. In addition, novel medication of heart failure, such as angiotensin receptor-neprilysin inhibitor (ARNI), has been proven to be associated with improved ejection fraction and prognosis (14); sodium-glucose cotransporter-2 inhibitors (SGLT2i) were effective in reducing cardiovascular and all-cause mortality in patients with heart failure with or without diabetes as well as improving cardiac function and LVEF especially in patients with HFrEF (32–34). Above all, medication up-titration and adherence are the principles of heart failure treatment. The study of Wang et al. indicated that up-titrating RASi and MRA were helpful in LVEF recovery as well as reverse ventricular remodeling, and Halliday et al. reported adverse LV remodeling upon therapy withdrawal in patients with heart failure with recovered LVEF. Discontinuation was another critical predictor of recurrence of left ventricular systolic dysfunction in patients with HFrecEF (35, 36). Therefore, individual up-titrated treatment, adherence to the guideline-directed management and therapy (GDMT), and the certification of optimal medical therapy (OMT), which included both medications and daily management of heart failure were essential for cardiac function improvement (37). However, there is still a lack of prospective data to guide the treatment of patients with improved LVEF or myocardial recovery, and there is little evidence for treatment strategy for patients with left ventricular ejection fraction in the borderline of 40–50% (HFmrEF) or complete recovery (left ventricular ejection fraction ≥ 50%) (38). Further investigation of the natural history and optimal treatment of such patients is therefore warranted.

To conclude, our study indicated that HFimpEF or HFrecEF is common among patients with heart failure with previously reduced ejection fraction, as approximately one-fifth of HFrEF would develop improved ejection fraction in the duration of the follow-up visit. HFimpEF reduces the risk of follow-up mortality and heart failure hospitalization to one-third compared with HFrEF with minor heterogeneity; therefore, follow-up EF monitoring is necessary to identify patients with HFimpEF for future risk assessment. For patients without HFimpEF, GDMT and up-titration for optimal medical therapy should be adopted to achieve improved ejection fraction. As the former studies reported that treatment cessation would lead to a reduction of EF (39), patients with improved EF should maintain the current treatment to avoid relapse.

Several limitations need to be acknowledged in our study. Firstly, the included studies had no unified definition of HFimpEF or HFrecEF, and there were also not enough articles or data for subgroup analysis. Therefore, it is uncertain which definition is associated with a better impact on prognosis. However, the articles included in this study clearly defined EF increase as the main criteria of HFimpEF, suggesting an increase in the impact of EF on prognosis. The impact of different definitions of HFimpEF on prognosis should be clarified through further research. Secondly, we have not obtained individual data from the included studies, so we cannot evaluate the adjusted effect of HFimpEF or HFrecEF on prognosis from all the included studies, which may cause a bias in the result. In addition, in the full-text review process, we found that some studies failed to provide valid effect size data of hazard ratio of HFrecEF on outcomes and therefore failed to get a more comprehensive assessment of HFrecEF for prognosis. Nonetheless, the studies included in our study were systematically searched and involved a large sample of patients with heart failure, which assured the rationality of conclusions for the pooled quantification of prognosis for patients with HFimpEF or HFrecEF. Finally, the studies we included were all observational, which aimed at exploring the relationship between the improvement of EF and prognosis. Further studies are necessary to pool the quantified effect of the intervention factors and risk factors on HFimpEF and the following outcomes.

## Conclusion

In this study, we performed a systematic review and meta-analysis to illustrate the prevalence and prognosis of HFimpEF who were developed from HFrEF. There were 22.64% of patients with HFrEF who would develop to HFimpEF in the duration of follow-up visit. For patients of HFimpEF, the risk of mortality would be reduced by 56 and 58% compared with HFrEF and HFpEF, respectively. In addition, HFimpEF was associated with a lower risk of heart failure hospitalization and composite events. Therefore, regular monitoring of EF is essential for heart failure patients during the follow-up visit. Aggressive treatments, such as guideline-directed medical therapy (GDMT) and optimal medical therapy (OMT), should be continued to achieve HFimpEF for patients with HFrEF. Further studies are required to explore how to improve the prognosis of patients with persistently reduced EF.

## Data Availability Statement

The deidentified participant data will be shared on a request basis. Please directly contact the corresponding author to request data sharing. All data relevant to the study are included in the article or uploaded as Supplementary Information.

## Author Contributions

YH, YoL, SC, and JC contributed to the study conception and design. WG, QL, SY, HH, RZ, ZG, JiaL, and LM contributed to literature search, study screening, and data extraction. YH and YiL contributed to the analysis and the first draft of the manuscript. SY, DL, JinL, and YY commented on and revised the final versions of the manuscript. All authors have read and approved the final manuscript.

## Funding

This study was funded and supported by the National Key Research and Development Program of China, Grant (No. 2016YFC1301202); Natural Science Foundation of Guangdong Province General Project (No. 2020A1515010940); and Guangdong Provincial Key Laboratory of Coronary Heart Disease Prevention (No. 2017B030314041).

## Conflict of Interest

The authors declare that the research was conducted in the absence of any commercial or financial relationships that could be construed as a potential conflict of interest.

## Publisher's Note

All claims expressed in this article are solely those of the authors and do not necessarily represent those of their affiliated organizations, or those of the publisher, the editors and the reviewers. Any product that may be evaluated in this article, or claim that may be made by its manufacturer, is not guaranteed or endorsed by the publisher.
