# Supplementary material for: Prevalence and Prognosis of HFimpEF Developed From Patients With Heart Failure With Reduced Ejection Fraction: Systematic Review and Meta-Analysis
Source: Front Cardiovasc Med. 2021 Nov 25;8:757596. doi: 10.3389/fcvm.2021.757596 (PMC8655693; doi:10.3389/fcvm.2021.757596)
Supplement: Supplementary file 1 [file Data_Sheet_1.docx]

Appendix: Literature search strategy

Cochrane Library

## improved NEAR/3 ejection fraction OR recovered NEAR/3 ejection fraction in Title Abstract Keyword AND heart failure OR heart dysfunction OR cardiac failure OR cardiac dysfunction in Title Abstract Keyword - (Word variations have been searched)

EMBASE

('heart failure':ab,ti OR 'heart dysfunction':ab,ti OR 'cardiac failure':ab,ti OR 'cardiac dysfunction':ab,ti) AND ('improved ejection fraction':ab,ti OR 'recovered ejection fraction':ab,ti)

Pubmed

((recovered ejection fraction[Title/Abstract]) OR (improved ejection fraction[Title/Abstract])) AND (((cardiac failure[Title/Abstract] OR cardiac dysfunction[Title/Abstract]) OR (heart failure[Title/Abstract] OR heart dysfunction[Title/Abstract])) OR (heart failure[MeSH Terms]))
